# The evolution of trends and technology in wearable sensors used to detect falls in people with neurodegenerative diseases: a systematic review

**DOI:** 10.3389/fnbot.2026.1766109

**Published:** 2026-03-13

**Authors:** Yuanzheng Chen, Tinghuai Huang, Zijie Lin, Quan Zhou

**Affiliations:** 1 Qingdao Preschool Education College, Qingdao, China; 2 Department of Rehabilitation Sciences, The Hong Kong Polytechnic University, Hong Kong SAR, China; 3 ShanTou No.1 Middle School, Shantou, China

**Keywords:** neurodegenerative diseases, wearable sensor, fall, fall detection algorithm, systematic review

## Abstract

**Background:**

Neurodegenerative diseases (NDs) are a significant threat to human health. Numerous research demonstrated that patients with NDs might present with decreased balance, which is responsible for an increased risk of falling. As an emerging technology, wearable devices can detect falls and prevent privacy breaches.

**Objective:**

To access the evolution of trends and technology in wearable devices to detect falls among patients with NDs.

**Methods:**

We screened PubMed and Web of Science (February 2023) to summarize the pathway of fall detection with any body-worn sensor. Included articles were required to be full-text and published in English. Documents were excluded if they; (1) only used wearable devices for fall cueing, (2) did not offer sufficient information for data extraction, (3) did not use patients with NDs, (4) only used non-wearable sensors or devices.

**Results:**

The review identified 89 articles at the end of the procedure for data extraction. A wide variety existed in participant sample size (1–131), sensor types, placement and algorithms. 97.75% of papers (*n* = 87) used patients with Parkinson’s disease as experimental subjects. 21.45% of studies attached devices on the ankle (*n* = 19), with a clear preference for using multiple types of sensors (58.43% of studies, *n* = 52). As the most commonly used inertial measurement unit (IMU), 21 articles utilized accelerometers and gyroscopes to assess falls. 39.33% of studies (*n* = 35) choose data set to verify the effectiveness of their algorithm. Machine learning algorithms have become prevalent since 2019, and the most commonly used algorithm was support vector machine (SVM) (*n* = 17).

**Conclusion:**

These results show that an increasing number of researchers examine the validation performance of their systems in non-real-time. The ankle was the preferred location among researchers, and there is a clear preference to use multiple types of sensors and machine learning algorithms to improve accuracy and immediacy. Future work should focus on other NDs instead of limiting to Parkinson’s disease and consider an adequately studied population. A consensus on walking tasks and accuracy measurements is urgently needed. Performing studies in a simulated free-living environment for a specified time frame is advisable, with continuous real-time monitoring and assessment.

**Systematic review registration:**

PROSPERO, identifier (CRD42023405952).

## Introduction

As a major threat to human health (Dommershuijsen et al., 2020; Kingwell, 2019), NDs (e.g., Parkinson’s disease, Alzheimer’s disease, motor neuron disease, and dementia) comprise a heterogeneous group of neurological conditions characterized by progressive—and often currently incurable—clinical courses. With the ongoing extension of lifespan, the prevalence and societal burden of these age-dependent disorders continue to rise (Heemels, 2016). Patients with NDs commonly exhibit motor impairments as well as cognitive and behavioral disturbances (Pender et al., 2020; Aarsland et al., 2021), which may manifest as impaired postural control, gait abnormalities (Morel et al., 2020), and consequently an elevated risk of falls (Schell et al., 2019). Falls in this population are not only associated with fractures, hospitalization, and loss of independence, but may also precipitate secondary complications (e.g., fear of falling and reduced mobility), thereby accelerating functional decline. Therefore, developing accurate and practical fall-detection solutions is of clinical importance to reduce injury-related morbidity and the downstream costs of post-fall care.

Wearable sensing has emerged as a promising approach for fall detection because sensors can be worn continuously and capture movement signals in everyday contexts when deployed at appropriate body locations. Compared with many environmental approaches, wearable solutions can support monitoring across both indoor and outdoor settings while offering a more privacy-preserving pathway for continuous assessment. Nevertheless, key barriers remain, including limited battery life, susceptibility to false alarms, and user adherence—factors that directly condition real-world feasibility even when laboratory performance is acceptable. Recent advances in mobile and embedded technologies have enabled miniaturized, energy-efficient devices with improved on-device processing and wireless connectivity, which can facilitate timely alerts and potentially mitigate adverse outcomes related to prolonged “long-lie” after a fall. Moreover, wearable platforms may function as personalized monitoring tools by providing quantitative, longitudinal information relevant to disease severity and mobility impairment, while reducing reliance on intrusive sensing modalities.

Despite substantial growth in the literature, fall-detection research in NDs populations remains methodologically heterogeneous, spanning diverse sensor modalities, placements, algorithms, and validation protocols, which contributes to fragmented evidence and limited cross-study comparability. Recent reviews in Ambient Assisted Living and Human Activity Recognition and wearable assisted-living have summarized broader wearable fall-detection advances and highlighted practical design constraints (e.g., unobtrusiveness, miniaturization, energy efficiency, and privacy) (Guerra et al., 2023; Li et al., 2025; Iadarola et al., 2024). Performance-oriented syntheses further underscore that validation performance is central to viability (Gorce and Jacquier-Bret, 2025). However, a NDs-focused synthesis that explicitly tracks how wearable fall detection has evolved over time—and that systematically compares validation performance across heterogeneous technological and methodological choices—remains limited. Accordingly, we conducted a systematic review to examine the temporal evolution of wearable-sensor fall detection in NDs populations in terms of sensor technology, body placement, algorithmic strategies, and validation performance, with the aim of clarifying robust evidence, improving comparability, and informing priorities for future investigations.

## Review methodology

A systematic literature review was conducted in light of the PRISMA statement (Liberati et al., 2009). We searched PubMed and Web of Science in February 2023 to summarize fall detection using body-worn sensors in patients with NDs. These databases were selected to allow both engineering and medical journals to be included during the search procedure. Additionally, a search in the reference of review articles and book chapters that appeared during the search was performed. The objective was to identify potentially eligible studies absent in the database search. The final search query is summarized in Table 1.

**Table 1:** Search string used for each database.

Database	Search string	Records
Web of Science	#1:(((((((((((((((((((((TS = (Parkinson*)) OR TS = (PD)) OR TS = (Paralysis Agitans)) OR TS = (Alzheimer)) OR TS = (ATD)) OR TS = (Dementia, Senile)) OR TS = (Senile Dementia)) OR TS = (Primary Senile Degenerative Dementia)) OR TS = (Dementia, Primary Senile Degenerative)) OR TS = (Dementia, Presenile)) OR TS = (Presenile Dementia)) OR TS = (Sclerosis, Amyotrophic Lateral)) OR TS = (ALS)) OR TS = (Gehrig’s Disease)) OR TS = (Gehrig Disease)) OR TS = (Gehrigs Disease)) OR TS = (Charcot Disease)) OR TS = (Guam Disease)) OR TS = (Disease, Guam)) OR TS = (motor neuron diseases)) OR TS = (Lou-Gehrigs Disease)) OR TS = (Disease, Lou-Gehrigs)	1,852
	#2: (TS = (fall*)) OR TI = (fall*)	
	#3: (((((TS = (sensor*)) OR TI = [13]) OR TS = (wearable*)) OR TI = (wearable*)) OR TS = (device*)) OR TI = (device*)	
	#1 AND #2 AND #3	
PubMed	#1: “parkinson*”[Title/Abstract] OR “PD”[Title/Abstract] OR “paralysis agitans”[Title/Abstract] OR “alzheimer*”[Title/Abstract] OR “ATD”[Title/Abstract] OR “dementia senile”[Title/Abstract] OR “senile dementia”[Title/Abstract] OR “primary senile degenerative dementia”[Title/Abstract] OR “dementia primary senile degenerative”[Title/Abstract] OR “dementia presenile”[Title/Abstract] OR “presenile dementia”[Title/Abstract] OR “amyotrophic lateral sclerosis”[Title/Abstract] OR “sclerosis amyotrophic lateral”[Title/Abstract] OR “ALS”[Title/Abstract] OR “motor neuron diseases”[Title/Abstract] OR “gehrig s disease”[Title/Abstract] OR “gehrig disease”[Title/Abstract] OR “charcot disease”[Title/Abstract] OR “guam disease”[Title/Abstract] OR “disease guam”[Title/Abstract]	484
	#2: “fall*”[Title/Abstract]	
	#3 “wearable*”[Title/Abstract] OR “sensor*”[Title/Abstract] OR “device”[Title/Abstract]	
	#1 AND #2 AND #3	

The truncation symbol was used to broaden the search with more specificity.

We included articles if they were full-text, published in English, and published in a peer-reviewed journal. In the meantime, involved papers should focus on fall detection or fall-risk assessment using wearable (body-worn) sensors in NDs populations, and present original research validating wearable sensors to assess falls or fall risk. We excluded articles if they; (1) only used wearable devices for fall cueing, (2) did not offer sufficient information for data extraction, (3) did not use patients with NDs, or (4) only used non-wearable sensors or devices. Algorithm performance metrics were not used as eligibility criteria; when reported, they were extracted and synthesized in the Results.

YC and TH finalized the standard of inclusion and exclusion, then independently screened the title, abstract and keyword in the databases. Repetitive outcomes were filtered out, and the remaining articles were relevant following their title and abstract. The remaining papers were reviewed in full document, and the applicable data was extracted from identified studies and tabularized under the pre-established heading. Divergences between reviewers were resolved by consensus. For each included study, we extracted the following variables: author(s), studied population, sensor type, device location(s) (including the number of placements, n), walking task, method category (threshold-based, machine learning, or deep learning), specific classifier/model, reported performance metrics, evaluation mode (online [ON] vs. offline [OFF]), publication year, real-time implementation (yes/no), and data source (e.g., public dataset vs. self-collected data).

## Results

### Studies selection

**Figure 1:**
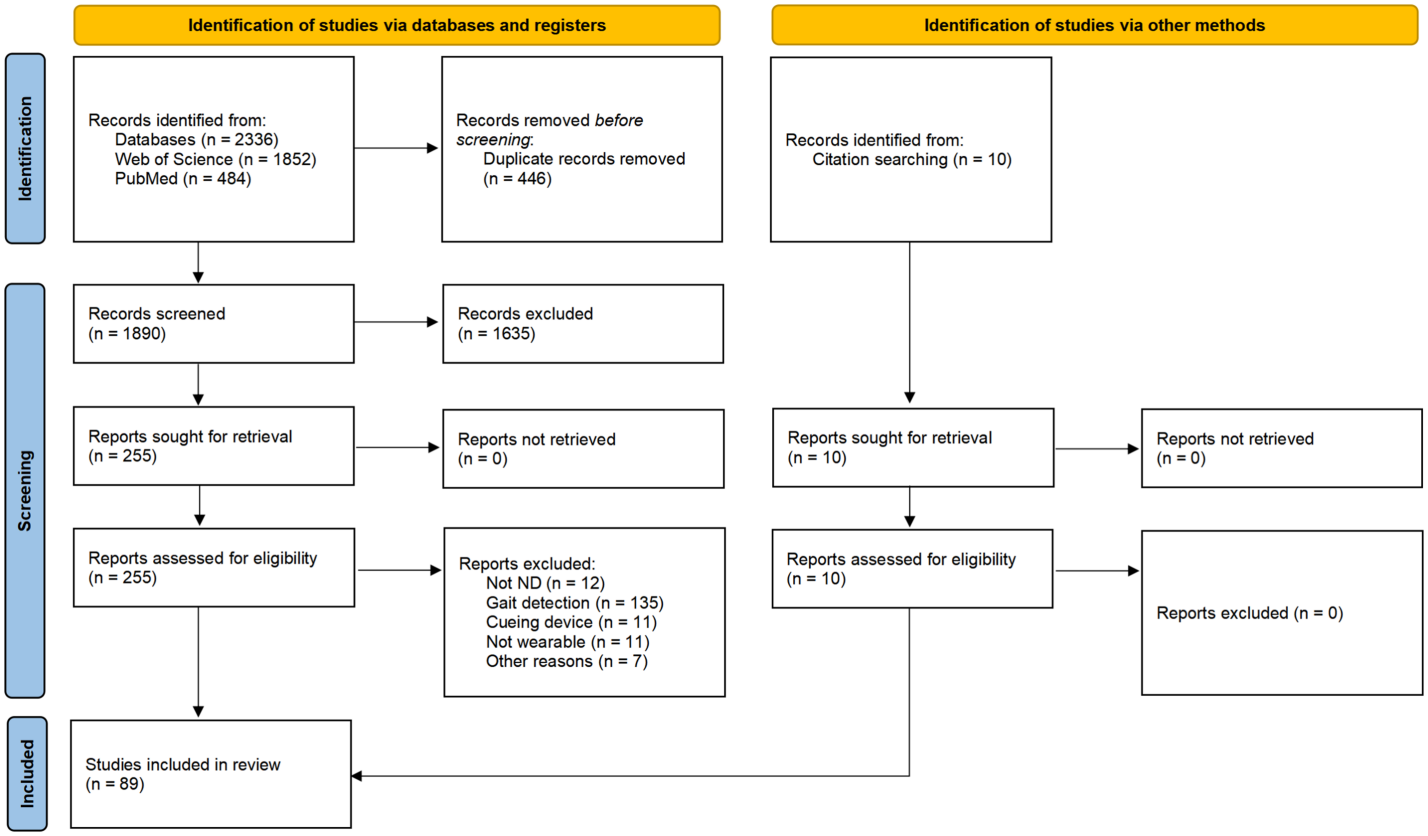
Study flow diagram.

The electronic database searches yielded 2,336 results that fulfilled the requirements for inclusion (Figure 1). Simultaneously, surveying the literature cited in these papers allowed for the identification of 10 more documents were included. Four hundred forty-six manuscripts were dismissed as duplicates, leaving 1890 papers being screened (1,635 records excluded). Of the remaining of 255 articles were filtered by full document. Eighty-nine articles were deemed relevant for this review.

**Figure 2:**
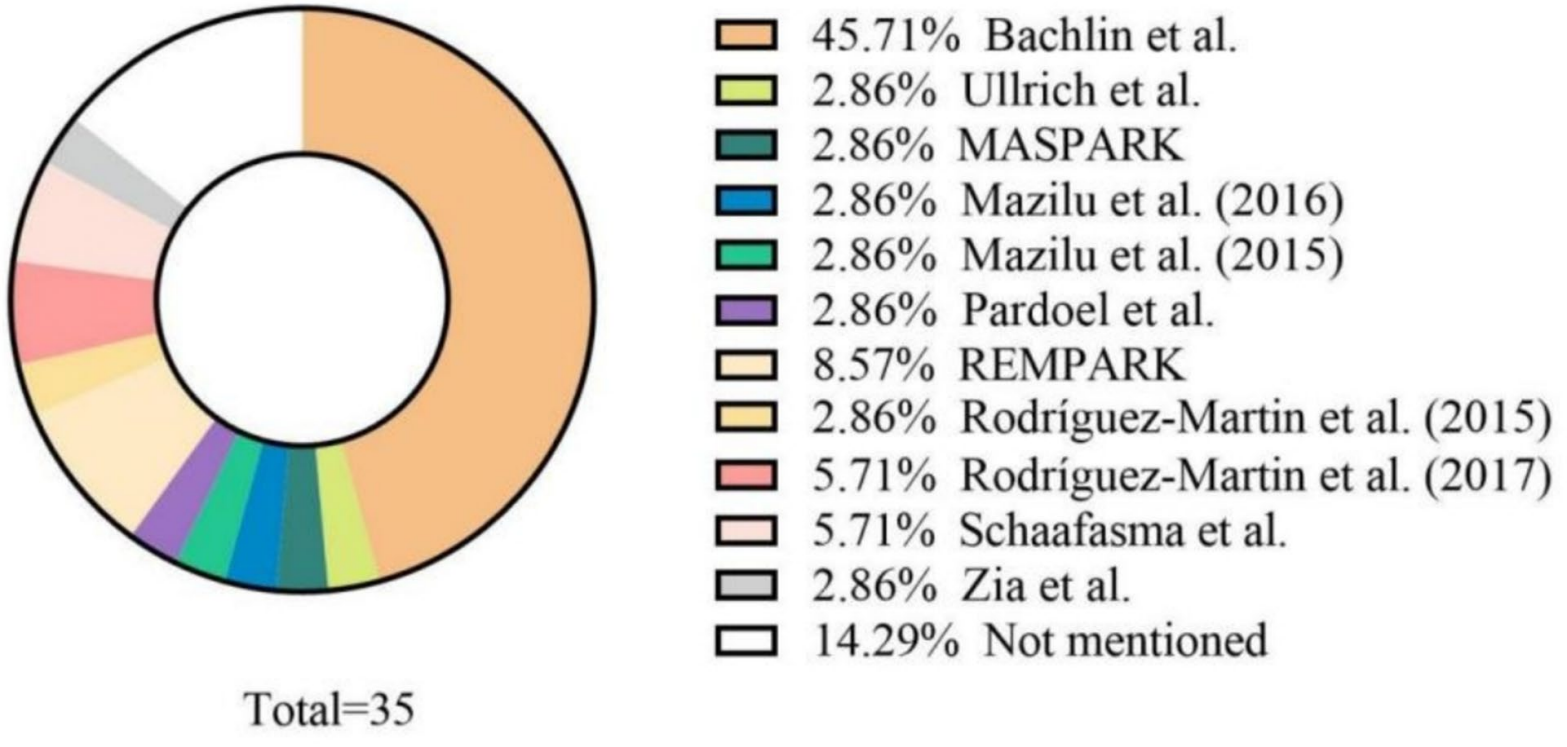
The source of data set.

This review analyzed the application of wearable sensors to access falls in patients with NDs (Table 2). Of all 89 articles, 87 articles used patients with Parkinson’s disease (PD), while seven and two articles recruited healthy elderly control and healthy control, and only one study enrolled neurological disorders sufferers. Concurrently, the enrollment count of fall detection projects ranged in complexity (range, 1–131, median = 14). Nevertheless, 39.33% of articles (*n* = 35) leverage data sets to appraise their algorithms’ credibility (Figure 2). Data from Bachlin et al. (2010) was the most frequently used data set (45.71% of studies, *n* = 16).

**Table 2:** Summary of fall detection studies.

Author	Studied population	Type of sensor	Device location (*n*)	Walking task	Method category	Classifier/model	Performance (reported metrics)	ON	OFF	Year	Real time	Source of dataset
Ahlrichs et al. (2016)	20 PD	Accelerometer; Gyroscope; Magnetometer	Waist (1)	Scripted activities simulating natural behavior at the patients’ home	Machine learning	SVM	Sensitivity: 92.3%; Specificity: 100.0%	–	–	2016	Y	Martín et al. (2015)
Ahn et al. (2017)	10 PD	Accelerometer; Gyroscope; Magnetometer	Head (1) Ankle (2)	TUG	Threshold	–	Accuracy: 92.86%	–	Y	2017	Y	–
Aich et al. (2018)	51 PD	Accelerometer	Knee (2)	Walking task	Machine learning	Naïve Bayes, SVM, k-NN, Decision Tree	Accuracy: 89.139%; Sensitivity: 88.524%; Specificity: 88.769%	–	–	2018	N	–
Arami (2019)	10 PD	Accelerometer	Lower back (1) Thigh (2) Shank (2)	Walking task	Deep learning	Probabilistic neural networks, SVM	Sensitivity: 93% (4); Specificity: 91% (6)	Y	–	2019	Y	Bachlin et al. (2010)
Cheng (2021)	10 PD	Accelerometer	Shank (1) Thigh (1) Lower back (1)	Unscripted and unconstrained activities of daily living in an apartment-like setting	Deep learning	LSTM, CNN	Window size of 3 Accuracy: 98.5%; Sensitivity: 98.5%; Specificity: 97.9% Window size of 4 Sensitivity: 96.9%; Specificity: 96.7%	–	–	2021	N	Bachlin et al. (2010)
Ayena et al. (2016)	7 PD 12 HC 10 HEC	Accelerometer	Sole (2)	The OLST at home as part of a serious game for balance training	Threshold	–	Discriminant validity: PD vs. non-PD OLST score(significant); The proposed OLST score has significantly differed between ground types	Y	–	2016	Y	–
Ayena and Otis (2020)	12 PD 9 HEC 10 HC	Accelerometer; Force sensor; Bending sensor	Sole (2)	TUG	Threshold	–	A significant difference was found for three FSR and IMU and on FSR and IMU in the elderly population (*p* < 0.001)	–	–	2020	N	–
Bikias et al. (2021)	11 PD	Accelerometer; Gyroscope	Wrist (1)	A series of walking task	Machine learning	Not specified	Leave-one-subject-out Sensitivity: 83%; Specificity: 88% Fold cross-validation Sensitivity: 86%; Specificity: 90%	–	–	2021	N	–
Borzì et al. (2021)	11 PD	Accelerometer; Gyroscope; Magnetometer	Shin (2)	TUG standardized 7-m course	Machine learning	SVM, LDA	The implemented classification algorithm in patients on Ayena and Otis (2020) therapy: Sensitivity: 84.1% (85.5%); Specificity: 85.9% (86.3%); Accuracy: 85.5% (86.1%) Machine learning: Sensitivity: 84.0% (56.6%); Specificity: 88.3% (92.5%); Accuracy: 87.4% (86.3%)	Y	Y	2021	Y	–
Borzi et al. (2019)	131 PD	Accelerometer; Gyroscope; Orientation sensor	FOG: waist (1) LA: thigh (1)	LA test; Unscripted and unconstrained activity of daily living	Machine learning	SVM, k-NN, neural network, decision tree, linear regression	LA test AUC: 92% FOG test AUC: 97%	–	–	2019	N	–
Camps et al. (2018)	21 PD	Accelerometer; Gyroscope; Magnetometer	Waist (1)	Walking task and dual task	Deep learning	CNN	Accuracy: 89.0%; Sensitivity: 91.9%; Specificity: 89.5%	Y	Y	2018	N	REMPARK
Capecci et al. (2016)	20 PD	Accelerometer	Hip (1)	TUG and dual task	Threshold	–	Moore-Bächlin Algorithm: Sensitivity: 70.10%; Specificity: 84.10% Moore-Bächlin Algorithm with step cadence: Sensitivity: 87.57%; Specificity: 94.97%	–	–	2016	Y	–
Charlon et al. (2013)	2 AD	Accelerometer	Upper back (1)	Free-living setting	Threshold	–	Sensitivity: 98.33%; Specificity: 97.77%	Y	–	2013	Y	–
Chomiak et al. (2019)	21 PD 9 HC	Accelerometer; Gyroscope	Above the patellofemoral joint line (1)	Walking task and dual task	Machine learning	–	Error rate: 0%; Sensitivity: 100%; Specificity: 100%	–	–	2019	Y	–
Coste et al. (2014)	4 PD	Accelerometer; Gyroscope; Magnetometer	Shank (1)	Walking task with dual tasking	Threshold	–	Sensitivity: 79.5%; Specificity: not reported; Only number of falls positives: 13 vs.35 true positives	–	–	2014	N	–
Cole (2011)	10 PD 2 HC	Accelerometer; Electromyographic	Forearm accelerometer (1) Thigh accelerometer (1) Skin accelerometer and Electromyographic (1)	Unscripted and unconstrained activities of daily living in an apartment-like setting	Machine learning	Dynamic neural network, linear classifier	Sensitivity: 82.9%; Specificity: 97.3%	–	–	2011	N	–
Demrozi et al. (2020)	10 PD	Accelerometer	Back (1) Hip (1) Ankle (1)	Walking task	Machine learning	k-NN	Sensitivity: 94.1%; Specificity: 97.1%	–	–	2020	Y	Bachlin et al. (2010)
Denk et al. (2022)	28 PD	Accelerometer; Gyroscope	Shoes (2)	A series of walking tasks	Threshold	–	Reported correlation: %TF (daily living) vs. %TF (in-home off-med testing) was mild-to-moderate; no correlation with on-med testing or self-report	Y	Y	2022	Y	–
Ghosh and Banerjee (2021)	10 PD	Accelerometer	Leg (2) Hip (2)	Walking task and dual task	Machine learning	LDA, CART, SVM, random forest	Accuracy: 89.94%; Sensitivity: 87.8%; Specificity: 93.02%	–	–	2021	N	Bachlin et al. (2010)
Djuric-Jovicic et al. (2014)	12 PD	Accelerometer; Gyroscope	Shank (2)	A series of walking tasks	Threshold	–	FOG-with-tremor accuracy: 100%; FOG-with-complete motor block accuracy: 100%; Normal stride accuracy: 95%; Short stride accuracy: 78%; Very short stride accuracy: 84%; Turning stride accuracy: 88%	–	Y	2014	N	–
Dvorani et al. (2021)	16 PD	Accelerometer	Shoe (2)	Walking task	Machine learning	SVM, AdaBoost classifiers	Sensitivity: 88.5% (5.8); Specificity: 83.3% (17.1); AUC: 92.8% (5.9)	–	–	2021	Y	–
El-Attar et al. (2021)	10 PD	Accelerometer	Ankle (1) Knee (1) Hip (1)	Walking task and dual task	Machine learning	SVM, artificial neural network	SVM accuracy: 87.5%; Neural network accuracy: 93.8%	–	–	2021	N	Bachlin et al. (2010)
Esfahani et al. (2021)	10 PD	Accelerometer; Gyroscope; Magnetometer	Shank (1) Thigh (1) Lower back (1)	Walking task	Deep learning	LSTM	Sensitivity: 92.57%; Specificity: 95.62%	–	–	2021	N	Bachlin et al. (2010)
Greene et al. (2018)	15 PD	Accelerometer; Gyroscope	Shank (2)	The free-living setting for 6 months	Threshold	––	Accuracy: 73.33%	–	–	2018	N	–
Guo et al. (2022)	12 PD	Electroencephalography	Waist on L5 (1) Leg (2)	Two TUG tasks	Deep learning	LSTM	Cross-subject setting GM: 91.0% (3.5); Subject-dependent setting GM: 91.0% (5.0)	–	Y	2022	N	–
Guo et al. (2019)	10 PD	Accelerometer	Ankle (1) Thigh (1) Hip (1)	Walking task and dual task	Machine learning	Time-varying autoregressive moving average model	Sensitivity: 99.20%; Specificity: 94.59%; Accuracy: (Average sensitivity: 96.86%, Specificity: 96.90%)	–	Y	2019	N	Bachlin et al. (2010)
Halder et al. (2021)	10 PD	Accelerometer	Ankle (1) Thigh (1) Hip (1)	Walking task and dual task	Machine learning	k-NN	FOG precision: 95.55% (4.60); Sensitivity: 94.97% (4.86); Specificity: 99.19% (0.85); F1 score: 95.25% (4.72); Accuracy: 98.92% (1.56); Pre of post FOG precision: 92.73% (10.15); Sensitivity: 91.5% (10.34); Specificity: 99.83% (0.32); F1-score: 92.10% (10.25)	–	–	2021	N	Bachlin et al. (2010)
Handojoseno et al. (2018)	16 PD	Electroencephalography	Head (1)	TUG on a standardized 5-m course	Machine learning	Optimal Bayesian neural network	Sensitivity: 85.86%; Specificity: 80.25%	–	–	2018	N	–
Iakovakis et al. (2016)	5 PD 10 HC	Sphygmomanometer; Smartwatch	Wrist (2)	Walking task	Machine learning	SVM, linear regression, neural network	Linear regression Predictive accuracy: 73%	–	–	2016	Y	–
Jovanov et al. (2009)	1 PD 4 HC	Accelerometer; Gyroscope	Knee (1)	Walking task	Threshold	–	Average detection latency: 332 ms (max 580 ms)	–	–	2009	Y	–
Iluz et al. (2014)	40 PD	Accelerometer; Gyroscope	Lower back (1)	Laboratory Walking tasks designed to provoke missteps; Home Participants worn the devices for 3 days during day time	Threshold	–	Criterion validity (lab) Hit ratio 93.1%; Specificity 98.6% Discriminant validity (home) Fallers vs. non-fallers odds ratio 1.84 (*p* = 0.01, 95% CI 1.15–2.93)	Y	Y	2014	N	–
Kim et al. (2015)	15 PD	Accelerometer; Gyroscope	Waist (1) Trouser pocket (1) Shin (1)	Walking task and dual (single) task	Machine learning	AdaBoost. M1 classifier	Waist sensitivity: 86.0%; Waist specificity: 91.7%; Pocket sensitivity: 84.0%; Pocket specificity: 92.5%	–	–	2015	N	–
Kim et al. (2018)	32 PD	Accelerometer; Gyroscope	In the trouser pocket (1)	A series of walking tasks	Deep learning	CNN	Sensitivity: 93.8%; Specificity: 90.1%	–	–	2018	N	–
Kita et al. (2017)	32 PD	Accelerometer; Gyroscope	Shin (2)	Walking task	Threshold	–	Specificity: 97.57%; Sensitivity: 93.41%; Precision: 89.55%; Accuracy: 97.56%	–	–	2017	N	–
Kwon et al. (2014)	20 PD	Accelerometer	Shoe (1)	Walking task	Threshold	–	Sensitivity: 86%; Specificity: 86%	Y	–	2014	N	–
Li et al. (2020)	10 PD	Accelerometer	Thigh (1) Calf (1) Lower back (1)	Walking task	Deep learning	LSTM	Sensitivity: 95.1%; Specificity: 98.8%	–	–	2020	N	Bachlin et al. (2010)
Kleanthous et al. (2020)	10 PD	Accelerometer	Ankle (1) Thigh (1) Trunk (1)	Walking task	Deep learning	Random forest, XGBoost, SVM, neural network	FOG sensitivity: 72.34%; FOG specificity: 87.36%; Transition sensitivity: 91.49%; Transition specificity: 88.51%; Normal activity sensitivity: 75.00%; specificity: 93.62%	–	Y	2020	N	Bachlin et al. (2010)
Mancini et al. (2021)	Study I: 45 PD Study II: 48 PD	Accelerometer; Gyroscope; Magnetometer	Study I: Shin (2) Foot (2) Wrist (2) Sternum and posterior trunk over L5 (1) Study II: Foot (2) Over the lumbar area (1)	Walking task	Threshold	Open-source algorithm	Rater 1: accuracy: 88%; sensitivity: 89%; specificity: 88%; false positive rate: 13%; false negative rate: 11%; AUC: 93% Rater 2: accuracy: 85%; sensitivity: 80%; specificity: 87%; false positive rate: 13%; false negative rate: 20%; AUC: 89%	–	Y	2021	N	–
Marcante et al. (2021)	20 PD	Accelerometer; Plantar pressure sensors	Sole (2)	A series of walking tasks	Threshold	–	Accuracy: 90%; False positive rate: 6%; False negative rate: 4%	Y	Y	2020	N	–
Masiala et al. (2019)	10 PD	Accelerometer	Thigh (1) Ankle (1) Lower back (1)	Walking task	Deep learning	Deep recurrent neural network, LSTM	Subject-independent: AUC 93%, Sensitivity 81%, Specificity 90%; Subject-dependent: AUC 97%, Sensitivity 87%, Specificity 96%	–	–	2019	N	Bachlin et al. (2010)
Mazilu et al. (2015a)	9 PD	Accelerometer	Ankle (2)	Free-living setting for 3 days	Machine learning	C4.5 pruned trees	(Performance NOT reported)	Y	–	2015	Y	–
Mazilu et al. (2016)	18 PD	Accelerometer; Gyroscope	Wrist (2) Ankle (2)	A series of walking task	Machine learning	Supervised machine learning	Subject-dependent accuracy: 85%; Specificity: 80%; Subject-independent accuracy: 90%; Specificity: 66%	Y	–	2016	Y	Mazilu et al. (2016)
Mazilu et al. (2015b)	18 PD	Electrocardiography; Skin-conductance	Chest (1) Finger (1)	Ziegler protocol, Cognitive tasks and hospital tour	Threshold	–	Predicting accuracy: 71.3% (4.2 s before episode)	Y	Y	2015	Y	–
Mazzetta et al. (2019)	7 PD	Accelerometer; Gyroscope; Magnetometer	Tibialis anterior (1) Gastrocnemius of the right leg (1)	TUG on standardized 7-m course	Threshold	–	False negative: 2%; False positive: 5%	Y	Y	2019	Y	–
Mesin et al. (2022)	12 PD	Accelerometer; Gyroscope; Electroencephalogram; Skin conductance; Electromyography; Electrocardiogram	Lateral tibia of the leg (2) Fifth lumbar spine (1) Wrist (1)	A series of walking task	Machine learning	SVM, k-NN	Subject-independent accuracy: 85%; Subject-dependent accuracy: 88%	–	Y	2022	N	?
Miko et al. (2019)	25 PD	IMU	Ankle (2)	TUG standardized 7-m course	Machine learning	Neural network	Sensitivity: 95.9%; Specificity: 93.1%	–	–	2019	Y	?
Moore et al. (2007)	11 PD 10 HC	Accelerometer	Shank (1)	A series of walking task	Threshold	–	Accuracy: 89%; Sensitivity: 89%; False positives: 10%	Y	Y	2008	N	–
Moore et al. (2013)	25 PD	Accelerometer	Lumbar region of the back (1) Thigh (2) Shank (2) Foot (2)	TUG tasks	Threshold	–	Lower back sensor, 10s window Sensitivity: 86.2%; Specificity: 82.4%	–	Y	2013	N	–
Naghavi et al. (2019)	18 PD	Accelerometer	Ankle (2)	A series of daily walking tasks	Machine learning	ADAptive SYNthetic sampling algorithm	Accuracy: 97.4%; Prediction: 66.7%	–	–	2019	Y	Schaafsma et al. (2003)
Naghavi and Wade (2019)	10 PD	Accelerometer	Shank (1) Thigh (1) Lower back (2)	Two walking tasks and one dual task	Threshold	–	Accuracy: 88.8%; Sensitivity: 92.5%; Specificity: 89.0%	–	Y	2019	Y	Bachlin et al. (2010)
Naghavi and Wade (2022)	7 PD	Accelerometer; Gyroscope	Ankle (2)	Walking task	Deep learning	CNN, transfer learning, k-means clustering	Sensitivity: 63.0%; Specificity: 98.6%; Target models identified 87.4% of FOG events, 21.9% predicted	–	–	2022	Y	Schaafsma et al. (2003)
O'Day et al. (2022)	16 PD	IMU	Chest (1) Lumbar region (1) Ankle (2) Feet (2)	Free-living setting	Deep learning	CNN	Lumbar and ankles AUROC: 83%	–	Y	2022	N	–
O'Day et al. (2020)	1 PD	IMU	Shank (2)	Walking task	Machine learning	–	(Performance NOT reported)	–	–	2020	Y	–
Palmerini et al. (2017)	18 PD	Electrocardiography; Skin-conductance	Shank (2) Lower back (1)	Walking task and dual task	Threshold	–	AUC: 76%; Sensitivity: 83%; Specificity: 67%	Y	–	2017	Y	Mazilu et al. (2015b)
Pardoel et al. (2022)	11 PD	Accelerometer; Plantar pressure sensors	Sole (2)	Walking task and dual task	Machine learning	Decision tree, Random undersampling boosting	Sensitivity: 77.3%; Specificity: 82.9%	–	–	2022	N	Pardoel et al. (2021b)
Pardoel et al. (2021a)	11 PD	Accelerometer; Gyroscope	Knee (2) Ankle (2)	Walking task along a complex pathway to provoke FOG	Threshold	–	Detection model episodes identified: 92.1% (8.2); precision: 31.8% (19.9); Prediction model episodes identified: 93.8% (6.8); precision: 30.6% (17.0)	Y	–	2021	N	–
Pardoel et al. (2021b)	11 PD	Accelerometer; Gyroscope; Plantar pressure sensor	Sole (2) Shank (2)	A series of walking task	Machine learning	Decision tree ensemble model	Total-FOG sensitivity: 76.4%, Specificity: 86.2%; Transition sensitivity: 85.2%; FOG sensitivity: 93.4%	–	Y	2021	Y	–
Pham et al. (2017)	10 PD	Accelerometer	Shank (1) Thigh (1) Lower back (1)	Walking task	Threshold	–	Sensitivity: 96%; Specificity: 79%; Ankle only Accuracy: 94%; Specificity: 84% Lower back only Accuracy: 89%; Specificity: 94%	–	Y	2017	N	Bachlin et al. (2010)
Pierleoni et al. (2019)	10 PD	Accelerometer; Gyroscope; Magnetometer	Chest (1)	Walking task	Threshold	–	Accuracy: 99.7%	–	–	2019	Y	–
Prado et al. (2021)	10 PD	Pressure sensor; Accelerometer; Angular velocity sensor; Euler angles sensor	Sole (2)	Zeno Walkway on a standardized 5-m course	Machine learning	Artificial neural network	Sensitivity: 96.0% (2.5); Specificity: 99.6% (0.3); Precision: 89.5% (5.9); Accuracy: 99.5% (0.4)	–	–	2021	Y	?
Prateek et al. (2018)	16 PD	Accelerometer; Gyroscope	Heel (2)	A series of walking task	Threshold	–	Accuracy: 81.03%	–	–	2018	N	–
Ly et al. (2017)	6 PD	Electroencephalography	Head (1)	A series of TUG	Deep learning	Bayesian neural network, Time-frequency Stockwell Transform	Sensitivity: 84.2%; Specificity: 88%; Accuracy: 86.2%	–	Y	2017	N	–
Reches et al. (2020)	71 PD	Accelerometer; Gyroscope; Magnetometer	Lower back (2) Ankle (2)	A series of walking tasks and dual task	Machine learning	SVM	Sensitivity: 84.1%; Specificity: 83.4%; Accuracy: 85.0%	Y	Y	2020	N	?
Ren et al. (2022)	12 PD	Accelerometer; Gyroscope; Plantar pressure sensor	Waist (1) Thigh (2) Shank (2) Sole (2)	Walking task	Threshold	–	Left-shank Sensitivity: 78.39%; Specificity: 91.66%; Accuracy: 88.09%; Precision: 77.58%; F-score: 77.98%	Y	–	2022	N	?
Rezvanian and Lockhart (2016)	10 PD	Accelerometer	Shank (1) Thigh (1) Lower back (1)	A series of walking task	Machine learning	CWT	Skin sensitivity: 84.9%; Specificity: 81.0% Thigh sensitivity: 73.6%; Specificity: 79.6% Lower back sensitivity: 83.5%; Specificity: 67.2%	Y	Y	2016	N	Bachlin et al. (2010)
Saad et al. (2017)	5 PD	Accelerometer; Telemeter; Goniometer	Shin (1)	Walking task	Machine learning	Gaussian neural network	Efficiency: 87%	–	–	2017	N	–
Ribeiro De Souza et al. (2022)	35 PD	Accelerometer; Gyroscope	Shank (1)	Turning trial	–	–	Turning trial: FoG ratio correlated with N-FoGQ score (significant); Total FoG time correlated with N-FoGQ (significant)	Y	–	2022	N	–
Sama et al. (2017)	15 PD	Accelerometer	Waist (1)	Walking task and dual task	Threshold	–	Sensitivity: 91.7%; Specificity: 87.4%	Y	Y	2018	Y	MASPARK project
Daniel et al. (2017)	12 PD	Accelerometer; Gyroscope	Waist (1)	Walking task, dual-task and free-living setting for 3 days	Machine learning	SVM	Sensitivity: 82.08%; Specificity: 93.75%	Y	Y	2017	Y	–
Rodríguez-Martín et al. (2017)	21 PD	Accelerometer	Waist (1)	A set of scripted activities at patients’ home	Machine learning	SVM	Generic model Sensitivity: 74.7%; Specificity: 79.0% Personalized model Sensitivity: 88.09%; Specificity: 80.09%	Y	Y	2017	Y	REMPARK project
Shalin et al. (2021)	11 PD	Accelerometer; Plantar pressure sensors	Sole (2)	Walking task	Deep learning	LSTM	Sensitivity: 82.1% (6.2); Specificity: 89.5% (3.6)	–	–	2021	Y	–
San-Segundo et al. (2019)	10 PD	Accelerometer	Ankle (1) Thigh (1) Lower back (1)	Walking task and dual task	Machine learning	Random forest, multilayer perceptron, Hidden Markov models	Sensitivity: 95%; Specificity: 75%	–	Y	2019	N	Bachlin et al. (2010)
Shi et al. (2022)	63 PD	Accelerometer; Gyroscope; Magnetometer	Ankle (2) 7th cervical vertebra of the spine (1)	TUG on standardized 7-m course and daily routine	Deep learning	CNN, CWT	GM: 90.7%; F1-score: 91.5%	–	–	2022	N	–
Shi et al. (2020)	67 PD	Accelerometer; Gyroscope; Magnetometer	Ankle (2) 7th cervical vertebra of the spine (1)	TUG on standardized 7-m course	Deep learning	CNN, CWT	Accuracy: 89.2%; GM: 88.8%	–	Y	2020	N	–
Sigcha et al. (2020)	21 PD	Accelerometer	Waist (1)	20 min of scripted ADL	Deep learning	Recurrent neural network	Sensitivity: 87.1%; Specificity: 87.1%; AUC: 93.9%	–	–	2020	N	Rodríguez-Martín et al. (2017)
Sigcha et al. (2022)	21 PD	Accelerometer	Waist (1)	Walking task and dual task	Deep learning	CNN	Sensitivity: 84.2%; Specificity: 93.9%; Precision: 61.7%	Y	Y	2022	N	Rodríguez-Martín et al. (2017)
Stamatakis et al. (2011)	1 PD 1 HC	Accelerometer	Hallux Heel (1) Foot (2)	Walking task	Threshold	–	(Performance NOT reported)	–	–	2011	N	–
Antonio et al. (2017)	44 PD	Accelerometer; Gyroscope	Shin (2)	TUG on standardized 3-m course	Threshold	*Ad hoc* algorithm	Accuracy: 98.51%; Sensitivity: 93.41%; Specificity: 98.51%; Positive predictive: 89.55%; Negative predictive: 97.31%	Y	Y	2017	N	–
Takac et al. (2013)	12 PD	Accelerometer; Gyroscope	Waist (1)	Walking task performed	Machine learning	Neural network	Root mean square error = 0.16	–	–	2013	Y	REMPARK project
Tamura (2005)	1 PD	Accelerometer	Chest (1)	Free-living setting	Threshold	–	Validity: Detected 19 of 22 falls (specificity/false positives not reported)	Y	Y	2005	N	–
Tang et al. (2020)	12 PD	Accelerometer; Gyroscope	Lower back (1)	TUG	Threshold	–	Sensitivity: 90.6% (7.71); Specificity: 94.3% (8.36)	–	–	2020	N	–
Tripoliti et al. (2013)	11 PD 5 HC	Accelerometer; Gyroscope	Wrist (2) Shin (2) Waist (1) Chest (1)	A series of walking tasks	Threshold	–	Sensitivity: 81.94%; Specificity: 98.74%	Y	Y	2013	N	–
Tzallas et al. (2014)	Lab: 24 PD Home: 12 PD	Accelerometer; Gyroscope	Wrist (2) Skin (2) Waist (1)	Lab: a series of walking tasks. Home: 5 consecutive days of free living.	Machine learning	Hidden Markov Model, SVM	Lab accuracy: 79%; Home error: 79%	Y	Y	2014	N	–
Ullrich et al. (2022)	40 PD	IMU	Shoe (2)	Free-living setting	Machine learning	Naïve Bayes, SVM, random forest, GBoost	Accuracy: 74%; Sensitivity: 60%; Specificity: 88%	–	–	2022	N	FallRiskPD dataset
Aner et al. (2014)	107 PD	Accelerometer	Lower back (1)	Patients wore the sensor for 3 consecutive days at home	–	–	Anterior–posterior width correlated with BBS (*r* = −0.30), DGI (*r* = −0.25), TUG (*r* = 0.32); In non-fallers, A-P width larger in fallers vs. non-fallers (*p* = 0.012)	Y	Y	2014	N	–
Xia (2018)	10 PD	Accelerometer	Shank (1) Thigh (1) Lower back (1)	Walking task	Machine learning	Adaboost algorithm, Random undersampling technique	Sensitivity: 99.70%; Specificity: 99.96%	–	–	2018	N	Bachlin et al. (2010)
Yungher et al. (2014)	14 PD	Accelerometer; Gyroscope; Magnetometer	Lower back (1) Thigh (2) Shin (2) Foot (2)	TUG on standardized 5-m course	Threshold	–	(Performance NOT reported)	–	Y	2014	N	–
Zach et al. (2015)	23 PD	Accelerometer	Waist (1)	Walking task	Threshold	–	Full rapid turns: Sensitivity: 78%, Specificity: 59%. Small steps: Sensitivity: 64%, Specificity: 69%. All tasks: Sensitivity: 75%, Specificity: 76%	–	Y	2015	N	–
Zia et al. (2021)	11 neurological disorder 12 HC	Accelerometer	Upper arm (1)	Free-living setting	Machine learning	Random forest, pruned decision tree, logistic model tree, Naïve Bayes, SVM	Forest stationary accuracy: 99.6%; Light ambulatory accuracy: 81.5%; Intense ambulatory accuracy: 97.2%	Y	–	2021	Y	Zia et al. (2020)

PD, Parkinson’s disease, AD, Alzheimer’s disease, FOG, freezing of gait, HEC, health elderly control, HC, healthy control, %TF, percentage of time spent frozen, LA, leg agility, BBT, Berg balance test, DGI, dynamic gait index, k-NN, k-nearest neighbor, SVM, support vector machine, LSTM, long short term memory, GM, geometric mean, NFoG-Q, new freezing of gait questionnaire, FSR, force sensitive resistor, IMU, inertial measurement unit, TUG, time up and go test, AUC, area under the curve, LDA, Linear discriminant analysis, CNN, convolutional neural network, CWT, Continuous wavelet transform, GBoost, Gradient Boosting, XGBoost, extreme Gradient boosting, CART, classification and regression trees, ADL, activity of daily living, hyphen signifies that articles used data set but did not provide the source of data set or cannot be found.

**Figure 3:**
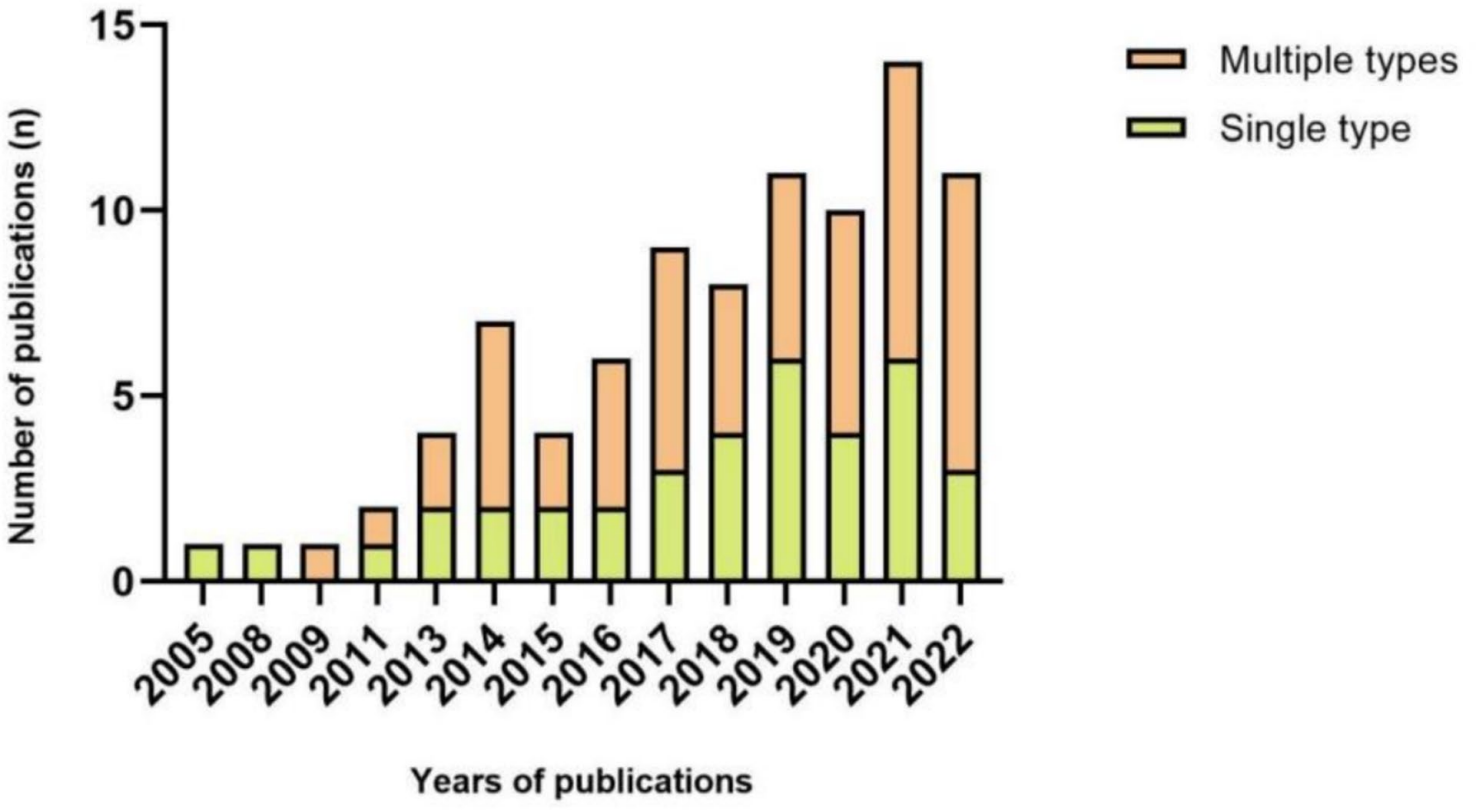
Number of publications each year per number of sensor type.

Studied employ a variety of sensor types and placements. From this review, we identified 37 papers that collected data with a single type of wearable sensor, including 33 projects that used an accelerometer alone, while one and three articles applied electroencephalography plantar pressure sensors. Fifty-two essays employed multiple forms of wearable sensors to assess falls (Figure 3). Twenty-one articles combined accelerometers and gyroscopes or along with magnetometers (*n* = 13). Since 2020, there has been a clear preference for using multiple devices to collect the activity data of the human body.

**Figure 4:**
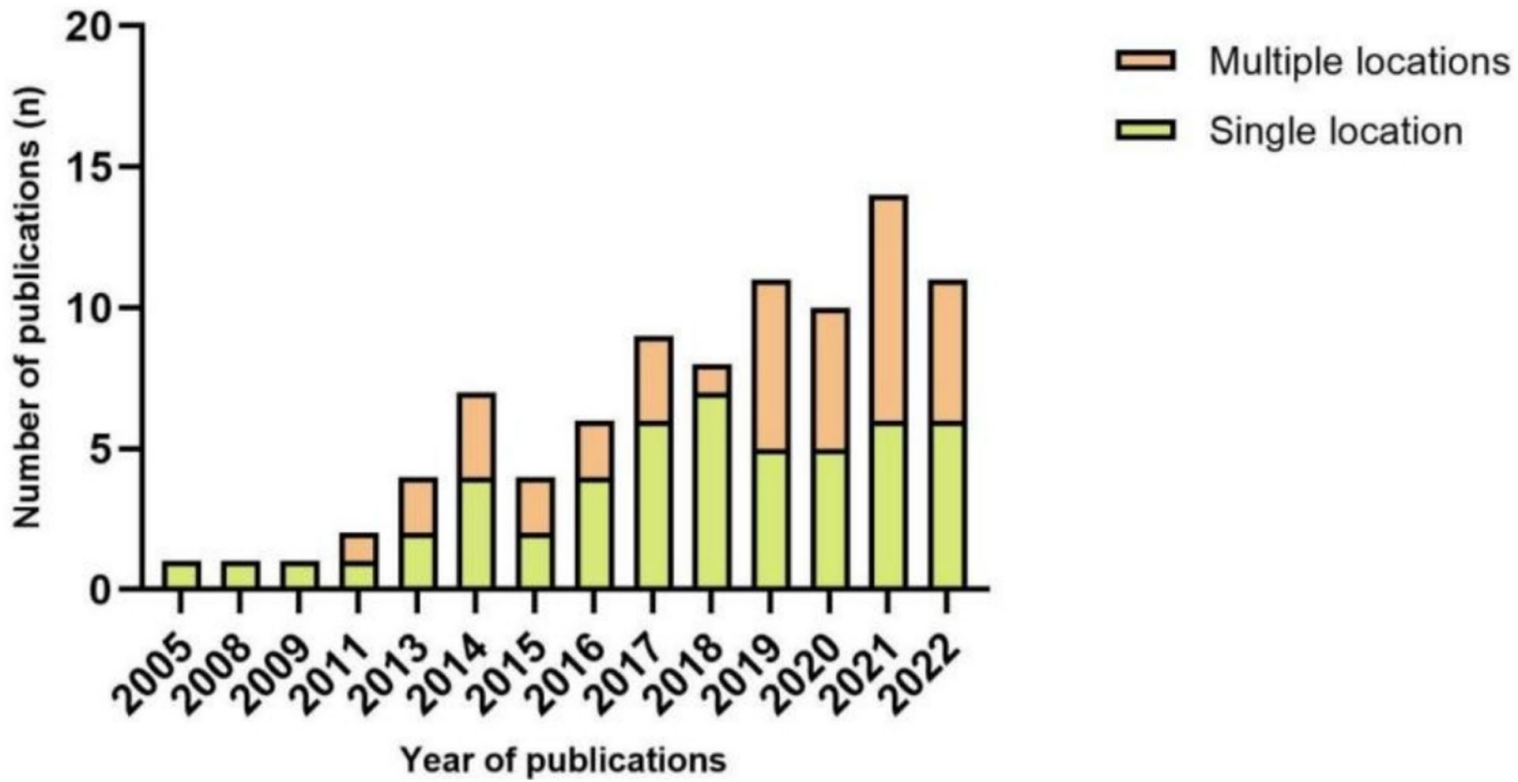
Number of publications each year per number of sensor locations.

Wearable sensors are positioned on different body regions to track physical activity (Figure 4; Table 3). The ankle was chosen as a sensor placement of 19 articles (12.10% of total placements; *N* = 157), with four studies using the ankle as the single placement site. Both the lower back and thigh were reported in 17 articles each (10.83% of total placements), and three studies adopted the lower back as the sole site. Shank and waist placements were reported in 16 (10.19%) and 15 (9.55%) articles, respectively, with five and ten studies using the shank and waist as the single placement sites. Notably, the “Ratio (%)” in Table 3 was calculated using the total number of device-location occurrences (total placements) as the denominator, rather than the number of included studies.

**Table 3:** Summary of device location of fall detection studies.

Body part	Body landmark or placement	Number of articles (*n*)	Ratio (%)	Single location (*n*)
Head and neck	Head	3	1.91	0
	7th cervical vertebra	2	1.27	0
Upper limb	Forearm	2	1.27	2
	Wrist	7	4.46	2
	Finger	1	0.64	0
Torso	Chest	5	3.18	2
	Upper back	2	1.27	1
	Lower back	17	10.83	3
	Lumbar	2	1.27	0
	Trunk	1	0.64	0
	Waist	15	9.55	10
Lower limb	Foot	5	3.18	0
	Gastrocnemius	1	0.64	0
	Hallux	1	0.64	0
	Heel	1	0.64	1
	Hip	6	3.82	1
	Knee	4	2.55	2
	Lateral tibia of leg	1	0.64	0
	Leg	1	0.64	0
	Sole	9	5.73	6
	Shank	16	10.19	5
	Shin	8	5.10	0
	Shoe	4	2.55	4
	Thigh	17	10.83	0
	Tibialis anterior	1	0.64	0
	Trouser pocket	2	1.27	1
	Ankle	19	12.10	4
	Patellofemoral joint line	1	0.64	1
Other	Skin	3	1.91	4

Ratio (%) was calculated using the total number of device-location occurrences included in this table (total placements, *N* = 157).

**Figure 5:**
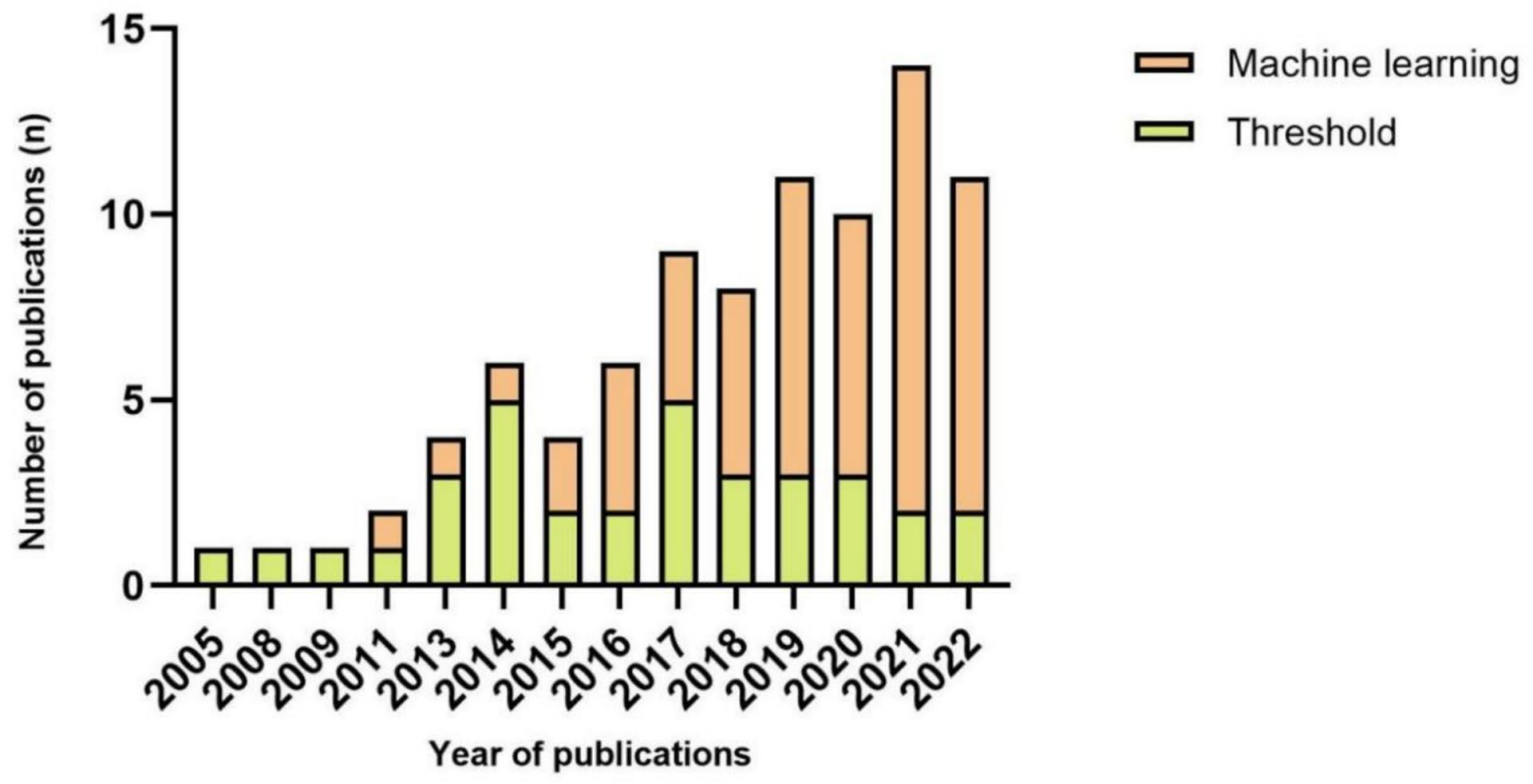
Number of publications each year per type of algorithm.

**Figure 6:**
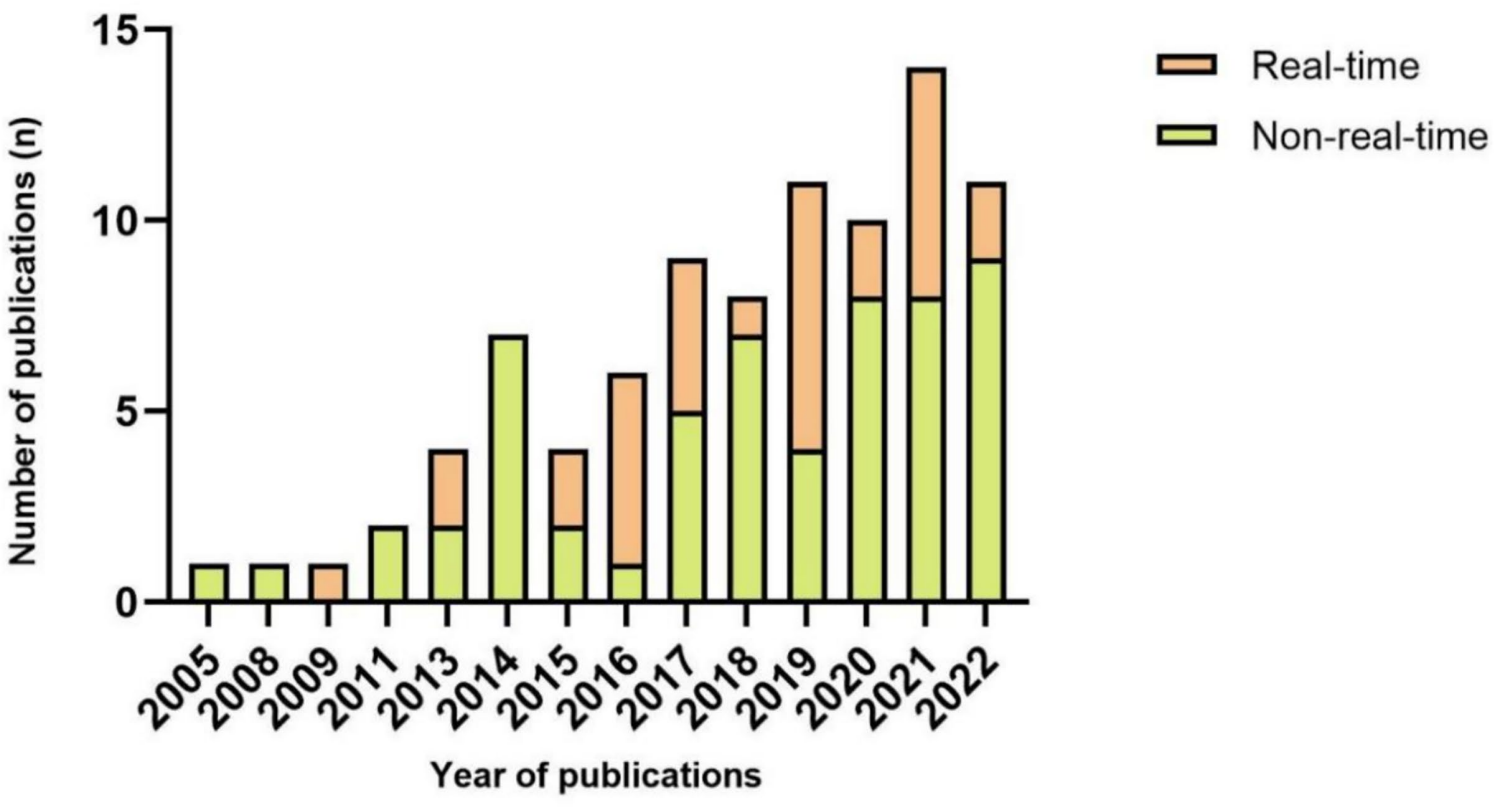
Number of publications each year per real-time evaluation.

The fall detection system heavily relies on the algorithm, which can range in complexity. Typically, threshold-based and machine learning-based are the two main types of algorithms in fall detection. A total of 34 articles relied on threshold-based algorithms for detecting falls, remaining 53 articles using machine learning. Figure 5 shows that the number of articles that used machine learning was two times higher than those that used threshold algorithms in 2019 and 2020, six times higher in 2021, and four times higher in 2022. Thirty-two articles opted for real-time evaluation as their preferred method. 62.5% of articles used machine learning algorithms to detect falls in real time (*n* = 20), and threshold algorithms were used in 12 of the 32 articles. Only in 2016 and 2019, the number of studies that detected falls in real-time was more significant than in non-real-time (Figure 6).

Among studies adopting machine learning/deep learning approaches (*n* = 53), SVM was the most frequently used classifier (*n* = 17, 32.07%), followed by CNN (*n* = 7, 13.20%) and LSTM (*n* = 6, 11.32%). Decision trees, neural networks, k-NN, and random forest were each reported in five studies (9.43%), whereas Naïve Bayes was used less frequently (*n* = 3, 5.66%). Reported performance varied across algorithms: k-NN achieved the highest median sensitivity (94.10%), whereas CNN yielded the highest median specificity (97.90%) (Table 4). The reported ranges also varied across models: SVM sensitivity ranged from 60.00 to 93.00% with specificity ranging from 80.09 to 100.00%, and random forest sensitivity ranged from 60.00 to 95.00% with specificity ranging from 75.00 to 93.02%. In addition, LSTM-based models reported relatively high median sensitivity and specificity (92.57 and 96.00%, respectively).

**Table 4:** Number of publications per type of machine learning algorithm.

Algorithm	Number of articles (n)	Ratio (%)	Sensitivity (%)	Specificity (%)
CNN	7	13.20	63.00–98.50 (MED = 89.00)	93.90–98.60 (MED = 97.90)
Decision trees	5	9.43	77.30–88.52 (MED = 82.91)	82.90–88.77 (MED = 85.83)
LSTM	6	11.32	82.10–98.50 (MED = 92.57)	89.50–97.90 (MED = 96.00)
Naïve Bayes	3	5.66	60.00–88.52 (MED = 74.26)	88.00–88.77 (MED = 88.38)
Neural network	5	9.43	72.34–95.90 (MED = 84.12)	87.36–93.10 (MED = 90.23)
SVM	17	32.07	60.00–93.00 (MED = 87.80)	80.09–100.00 (MED = 80.30)
k-NN	5	9.43	88.52–94.97 (MED = 94.10)	88.77–99.83 (MED = 97.10)
Random forest	5	9.43	60.00–95.00 (MED = 80.07)	75.00–93.02 (MED = 87.68)

CNN, convolutional neural network, LSTM, long short term memory, SVM, support vector machine, k-NN, k-nearest neighbor, MED, median, Ratio (%) was calculated using the number of studies included in this table (*N* = 53).

Studies exhibit remarkable diversity in the measures of validation performance (Table 5). Overall, the reviewed studies had a median sensitivity of 88.09% (range, 60–100%), a median specificity of 92.08% (range, 67–100%), and a median accuracy of 89.57% (range, 71.3–100.0%).

**Table 5:** Number of publications per type of outcome for each device combination.

Combination	Number of articles (*n*)	Ratio (%)	Sensitivity (%)	Specificity (%)
Accelerometer, gyroscope and magnetometer	13	25.00	79.50–92.57 (MED = 89.00)	83.40–100.00 (MED = 88.90)
Accelerometer, force sensor and bending sensor	1	1.92	–	–
Accelerometer and gyroscope	21	40.38	63.00–100.00 (MED = 88.30)	80.00–100.00 (MED = 97.57)
Accelerometer, gyroscope and orientation sensor	1	1.92	–	–
Accelerometer and electromyographic	1	1.92	82.90	97.30
Sphygmomanometer and smartwatch	1	1.92	–	–
Accelerometer and plantar pressure sensors	3	5.77	82.10	89.50
Electrocardiography and skin-conductance	2	3.85	83.00	67.00
Accelerometer, gyroscope, electroencephalogram, skin conductance, electromyography, electrocardiogram	1	1.92	–	–
IMU	4	7.69	60.00–95.90 (MED = 77.95)	88.00–93.10 (MED = 90.55)
Accelerometer, gyroscope and plantar pressure sensors	2	3.85	78.39	91.66
Pressure sensors, accelerometer, angular velocity sensor and Euler angles sensor	1	1.92	96.00	99.60
Accelerometer, telemeter and goniometer	1	1.92	–	–

IMU, inertial measurement unit, MED, median, Ratio (%) was calculated using the number of studies included in this table (*N* = 52).

## Discussion

This systematic review sought to explore the literature on fall detection and determine the mainstream sensor type, device location, sources of dataset, and algorithm. This paper also elucidated the evolution of trends and technology. In all, 89 articles were analyzed in this review.

### Falls detection apparatus

Several categories of all detection apparatus include vision-based, wearable device-based and ambient-based approaches (Mubashir et al., 2013). Owing to the evolution of wireless signal transmission and electronic miniaturization technology, numerous studies are investigating the effectiveness of wearable sensors for fall detection. In this review, sensor types and combinations exhibited significant diversity between studies. In 37 studies, fall detection was carried out using only one type of wearable sensor. Accelerometers alone were used in 89.2% of the studies (n = 33), whose sensitivity ranged from 72.34 to 99.70% (MED = 88.50%) and specificity ranged from 75.00 to 99.96% (MED = 88.88%). Three articles exclusively relied on electroencephalography for fall detection (sensitivity: 77.30–85.86%, specificity: 80.25–88.00%), and one article used the pressure sensor alone (Pardoel et al., 2022). These findings suggest that fall detection accuracy remains consistent regardless of the number of sensor types. Using a single sensor type can streamline the computing demands and sophistication of the system.

A considerable percentage of studies on fall detection utilized IMU, which included more than one type of sensor. Among the identified papers, IMU can found to be utilized for fall detection in 52 of them, only 3 of them exclusively mentioned IMU, whereas the other 49 articles described the type of sensors used. This review revealed that the most commonly used sensor combination in fall detection studies was the integration of an accelerometer and a gyroscope (*n* = 21). Furthermore, there was only a slight difference in accuracy between different sensor combinations. The only exception to this finding was the specificity of integrating electrocardiography and skin conductance (67%).

The path of trend in fall detection can be attributed to several factors. Firstly, IMUs can collect data from multiple axes to capture the full range of body movement during a fall event, improving accuracy. Second, electronic miniaturization technology can minimize energy consumption and chip size while maintaining high performance, which makes IMUs more accessible and affordable for developers to use in fall detection. Finally, researchers can analyze large datasets and derive accurate conclusions since machine learning-based tools are increasingly becoming available.

### Sensor placement

As previously mentioned, multiple protocols were outlined for fall detection devices to access falls. The human body has four divisions: the head and neck, the torso, the upper limb, and the lower limb. The lower limb was the predominant placement, accounting for 76.49% of the studies (*n* = 68). The ankle was the preferred location among researchers (21.34% of studies, *n* = 19), and the sole was the most frequently selected single location on the lower limb (21.34% of studies, *n* = 19). The waist was both the most frequent location on the torso and the most common single placement on the human body (*n* = 10). As a crucial weight-bearing structure, the lower limb can intuitively reflect the impact experienced by users and is therefore an essential consideration in clinical assessments. Placement-specific performance comparisons could not be synthesized reliably, as many included studies deployed multiple sensor locations yet reported only aggregate performance without location-stratified results. Future primary studies should report location-specific performance in multi-placement designs or conduct head-to-head comparisons under standardized sensor combinations and protocols to enable robust placement-based meta-analyses.

### Algorithms

The most straightforward approach for fall detection is the threshold-based algorithm. Among the articles identified in this review, 34 employed threshold-based algorithms for fall detection. With threshold-based algorithms, a fall is detected if chosen indicators exceed a selected threshold. Falls can not be detected as having happened unless the criteria are met. With optimized computational performance, threshold approaches can conduct a rapid analysis of massive data. Still, plenty of drawbacks exist in threshold-based algorithms. A strict threshold may reduce the probability of detecting falls, and a loose threshold may increase the likelihood of detecting false positives. This is a situation that most investigators find themselves in.

Machine learning algorithms such as convolutional neural networks (Cheng, 2021; Camps et al., 2018; Kim et al., 2018; Naghavi and Wade, 2022; O'Day et al., 2022; Shi et al., 2022; Shi et al., 2020; Sigcha et al., 2022), decision trees (Aich et al., 2018; Borzi et al., 2019; Pardoel et al., 2022; Pardoel et al., 2021b), long short term memory (Cheng, 2021; Esfahani et al., 2021; Guo et al., 2022; Li et al., 2020; Masiala et al., 2019; Shalin et al., 2021) (*n* = 6), Naïve Bayes (Aich et al., 2018; Ullrich et al., 2022; Zia et al., 2021), neural network (Borzi et al., 2019; Iakovakis et al., 2016; Kleanthous et al., 2020; Miko et al., 2019; Takac et al., 2013), SVM (Aich et al., 2018; Ahlrichs et al., 2016; Arami, 2019; Borzì et al., 2021; Borzi et al., 2019; Ghosh and Banerjee, 2021; Dvorani et al., 2021; El-Attar et al., 2021; Iakovakis et al., 2016; Kleanthous et al., 2020; Mesin et al., 2022; Reches et al., 2020; Daniel et al., 2017; Rodríguez-Martín et al., 2017; Tzallas et al., 2014; Ullrich et al., 2022; Zia et al., 2021) (*n* = 17), k-nearest neighbor (Aich et al., 2018; Borzi et al., 2019; Demrozi et al., 2020; Halder et al., 2021; Mesin et al., 2022), and random forest (Ghosh and Banerjee, 2021; Kleanthous et al., 2020; San-Segundo et al., 2019; Ullrich et al., 2022; Zia et al., 2021) were used extensively in recent studies to address limitations of threshold-based approaches, particularly the need for manual threshold selection and potential sensitivity to inter-individual variability.

As summarized in Table 4, a range of machine learning and deep learning classifiers has been adopted; however, drawing algorithm-level conclusions remains difficult because reported performance is strongly dependent on sensor configuration, placement, task protocol, and validation design. Wearable devices were deployed for data collection, and a training phase is an integral part of machine learning. The traditional understanding is that machine learning algorithms are more computationally demanding than threshold-based methods, resulting in higher latency (Hu and Qu, 2016). Nevertheless, more and more studies used machine learning algorithms to detect falls in real time. This trend suggests that machine learning is increasingly feasible for on-device or near-real-time deployment and is emerging as a leading strategy to improve the reliability of fall detection systems (Figure 5).

To really bring fall detection into practical use, a significant obstacle still resides in evaluation in real-time. Theoretically, with the rapidly improving computational capability of CPU, the difficulty of assessment in real-time can be reduced. However, there is no association between publication year and the number of studies that evaluate the activity data of the human body in real-time. The above can be attributed to the current studies intended to investigate datasets to assess the effectiveness of their algorithms, improving productivity and conserving resource usage.

### Fall detection performance

Multiple performance metrics can be used to assess the reliability of fall detection systems, including sensitivity, specificity, accuracy and so on. Sensitivity was a commonly used performance metric in 59.55% of studies, with a wide range of sensitivity values from 60 to 100%. The sensitivity of Chomiak et al. (2019) was perfect, achieving a sensitivity of 100%. The lowest sensitivity value of 60% was obtained by Ullrich et al. (2022). Meanwhile, the exact number of papers used specificity (59.55% of studies, *n* = 53), with specificity values ranging from 67 to 100%. The approach of Palmerini et al. (2017) yielded the lowest specificity of 67%, and two articles reported 100% specificity (Ahlrichs et al., 2016; Chomiak et al., 2019). Some articles utilized accuracy as a performance metric, with an accuracy range of 71.3–100.0%. The fall detection system proposed by Djuric-Jovicic et al. (2014) exhibited the highest accuracy (100%), and the lowest accuracy was obtained by Mazilu et al. (2015b). A few studies reported AUC varying from 76 to 97%, with the highest AUC values achieved by two articles (Borzi et al., 2019; Masiala et al., 2019) and Palmerini et al. (2017) had the lowest value. Meanwhile, some measures of validation performance were utilized in a few studies, such as f-score (*n* = 2), geometric mean (*n* = 3), error rate (*n* = 1), false positive rate (*n* = 3), false negative rate (*n* = 2), positive/negative predictive (*n* = 1), root mean square error (*n* = 1), mean absolute error (*n* = 1).

Of note, it is challenging to draw a firm conclusion on the optimal fall detection system based solely on the reported validation performance, due to substantial heterogeneity across the included studies. The evidence base is population-imbalanced and often underpowered (87/89 studies on Parkinson’s disease; sample size 1–131, median = 14), while sensor configurations and placements vary widely (including multi-sensor and multi-location designs), limiting attribution of performance to any single design choice. In addition, protocols and evaluation settings differ (task definitions; offline vs. online/real-time testing), and studies use heterogeneous data sources and reporting practices, with wide ranges in sensitivity, specificity, and accuracy. These factors confound direct comparisons and prevent identification of a single “preferred” solution for NDs populations. A standardized benchmarking and reporting framework is urgently needed for fair cross-study comparisons. To improve cross-study comparability, future work should adopt harmonized reporting of participant characteristics, sensor configurations/placements, and evaluation protocols/settings, alongside a core set of performance metrics. Prospective, adequately powered studies across broader NDs phenotypes are also needed to validate deployment-ready systems under real-world conditions.

### Conclusion and future work

Affordable, efficient healthcare for patients with NDs is eminently needed. Through an examination of 89 articles on wearable sensors for fall detection, this review provided a comprehensive overview of the evolution of trends and technology in this area. Various aspects were examined in this paper, including sensor type utilized, device placement, the number of subjects (datasets) considered, algorithms implemented, and validation performance achieved. More and more studies have embraced machine learning algorithms to improve the accuracy and immediacy of fall detection systems, thanks to the enhancement of computing capacity power. Furthermore, there is a clear preference to use multiple types of sensors to detect falls. Despite evaluation in real-time being a critical step to put fall detection into practical use, an increasing number of researchers examine the validation performance of their systems in non-real-time. Many investigators targeted their attention to patients with Parkinson’s disease and ignored other NDs. The number of study participants was limited, and a consensus has not been reached on a standard walking test, which might create difficulties for researchers trying to find the optimal system according to the reported validation performance. Furthermore, there is an absence of agreed-upon machine learning algorithms. Future work must address the limitations highlighted in this research to advance the field. Firstly, the studied population should be carefully selected to support their viewpoints, and more attention should be given to other NDs. Secondly, a consensus on walking tasks and accuracy measurements is urgently needed. Lastly, with continuous real-time monitoring and assessment, performing studies in a simulated free-living environment for a specified time frame is advisable.

## Limitations

The major limitation of this systematic review is the limited number of papers included in this review. Due to the search strategy, many related documents written in other languages and electronic databases may have been omitted. Furthermore, manual screening and review procedures may lead to a potential loss of papers and be subject to interpretive bias.

## Data Availability

The original contributions presented in the study are included in the article/supplementary material, further inquiries can be directed to the corresponding author.
